# Nanotechnology in Pain Management

**DOI:** 10.3390/pharmaceutics16111479

**Published:** 2024-11-20

**Authors:** Andrew Torpey, Emily Bellow, Veronica Samojedny, Sukhpreet Ahluwalia, Amruta Desai, William Caldwell, Sergio Bergese

**Affiliations:** 1 Department of Anesthesiology, Stony Brook University Hospital, Stony Brook, NY 11794, USA; andrew.torpey@stonybrookmedicine.edu (A.T.); amruta.desai@stonybrookmedicine.edu (A.D.); william.caldwell@stonybrookmedicine.edu (W.C.); 2 Renaissance School of Medicine, Stony Brook University, Stony Brook, NY 11794, USA; emily.bellow@stonybrookmedicine.edu (E.B.); veronica.samojedny@stonybrookmedicine.edu (V.S.); 3 Department of Surgery, Stony Brook University Hospital, Stony Brook, NY 11794, USA; sukhpreet.ahluwalia@stonybrookmedicine.edu; 4 Department of Neurosurgery, Stony Brook University Hospital, Stony Brook, NY 11794, USA

**Keywords:** nanotechnology, chronic pain, inflammation, drug delivery, nanomaterials

## Abstract

Chronic pain is a debilitating condition that affects millions of patients worldwide, contributing to a high disease burden and millions of dollars in lost wages, missed workdays, and healthcare costs. Opioids, NSAIDs, acetaminophen, gabapentinoids, muscle relaxants, anticonvulsants, and antidepressants are the most used medications for chronic pain and carry significant side effects, including gastric bleeding, hepatotoxicity, stroke, kidney damage, constipation, dizziness, and arrhythmias. Opioids in particular carry the risk of long-term dependence, drug tolerance, and overdose. In 2022, 81,806 people died from opioid overdose in the United States alone. Alternative treatments for chronic pain are critically needed, and nanotechnology has emerged as a promising means of achieving effective long-term analgesia while avoiding the adverse side effects associated with conventional pharmacological agents. Nanotechnology-based treatments include liposomes, Poly Lactic-co-Glycolic Acid (PLGA) and other polymeric nanoparticles, and carbon-based polymers, which can help mitigate those adverse side effects. These nanomaterials can serve as drug delivery systems that facilitate controlled release and drug stability via the encapsulation of free molecules and protein-based drugs, leading to longer-lasting analgesia and minimizing side effects. In this review, we examine the role of nanotechnology in addressing concerns associated with conventional chronic pain treatments and discuss the ongoing efforts to develop novel, nanotechnology-based treatments for chronic pain such as nanocapacitor patches, gene therapy, the use of both viral and non-viral vectors, CRISPR, and scavengers.

## 1. Introduction

Chronic pain is defined as pain on most days or every day persisting for over 3 months, and it is a public health problem affecting millions of people worldwide. In 2019, an estimated 20.5% of adults in the United States suffered from chronic pain, highlighting the high disease burden [1]. Non-chronic pain, or acute pain, is also highly prevalent, and millions more Americans experience acute pain conditions related to surgical procedures, illness, or accidents. A significant portion (14.9%) of those reporting non-chronic pain reported progressing to chronic pain one year later [2]. Annually, the estimated cost of pain is estimated to be from $560 billion to $635 billion, including direct healthcare costs, lower wages, and work missed [3].

Opioids have long been used for the management of pain. Morphine was first isolated from opium in 1803 and during the 20th century new opioids, including oxycodone and hydromorphone, were developed [4]. Opioids are effective at reducing pain. Patients suffering from chronic pain who received opioid medications had significantly more pain relief compared to patients who received a placebo. Strong opioids, including morphine and oxycodone, were also found to be significantly more effective for pain relief when compared to non-steroidal anti-inflammatory drugs [5]. However, their long-term utilization is linked to many multi-system side effects, as well as the potential for opioid abuse, addiction, and overdose. The opioid epidemic has had far-reaching effects on the economy, health, and wellbeing of communities across the country. In 2022, 81,806 people died from opioid related overdoses in the U.S [6]. In addition to the large number of fatal overdoses, an estimated 7 million Americans struggle with opioid use disorder (OUD). The crisis is associated with the reduction in life expectancy, with 3.1 million years of life estimated to be lost due to opioid overdoses in 2022 alone [6]. Further negative effects of opioids include side effects such as constipation, nausea, drowsiness, fractures, and cardiovascular/respiratory events [5,7].

Other multimodal medications including NSAIDs, acetaminophen, gabapentinoids, muscle relaxants, and antidepressants have also been used in the treatment of pain. Their side effects are also profound and include gastric bleeding, stroke, kidney damage, constipation, dizziness, and arrhythmias [8,9]. The widespread prevalence of pain and the significant side effects associated with current pain medications underscore the urgent need to explore alternative treatment modalities.

Based on a comprehensive review, a search has been conducted for the words nanotechnology, nanomaterials, drug delivery, chronic pain, inflammation, and nanocapacitor patches, in various databases, such as PubMed, Science Direct, Google Scholar, and Cochrane library. Patents and articles, which were found to be relevant and have up-to-date information regarding the achievement of long-term analgesia and reduction in side effects from common analgesics, have been selected for inclusion.

## 2. Role of Nanotechnology

Despite their efficacy in achieving analgesia, opioids are not universally well-tolerated by patients, nor are they able to achieve targeted pain relief [10]. In particular, the development of patient tolerance and dependence/addiction to opioids is a concern, and thus the development of nanoparticulate systems that address this problem has been a priority. In addition to the addiction potential associated with opioid use, NSAIDs also carry the risk of numerous unwanted side effects, including hepatoxicity, gastric bleeding, myocardial infarction, stroke, peptic ulcers, and kidney damage [8]. These side effects directly result from the NSAID mechanism of action, which is cyclooxygenase inhibition; cyclooxygenases are critical for optimizing various physiologic functions throughout the body [11].

With the goal of avoiding the systemic side effects that accompany cyclooxygenase inhibition, topical nanomaterials such as NeuroCuple^TM^ have been designed as analgesic alternatives. NeuroCuple^TM^ is a nanocapacitor-based patch that does not contain any chemicals or require an energy source. A randomized control study of 69 patients who were assigned to receive either NeuroCuple^TM^ or the standard of care after unilateral total hip and knee arthroplasty showed that patients in the NeuroCuple^TM^ group had a significant 34% reduction in pain at rest, experienced a 9% reduction in in-hospital opioid consumption, and were significantly less likely (26% vs. 55%) to request an opioid prescription upon discharge from the hospital [12]. In addition to effective analgesia, which is an important component of high-quality post-operative care, this study also demonstrates the potential role of nanotechnology in preventing postoperative opioid addiction. Over 80% of patients receive opioids after low-risk surgery, and over 80% of those prescriptions include hydrocodone or oxycodone, the two opioids most frequently implicated in overdose death [13]. Surgical patients are therefore a critically important group that must be addressed in the search for safer, more effective analgesic methods, and nanotechnology demonstrates significant potential to address the post-operative needs of this patient population.

Complementary and alternative medicine approaches have grown in popularity over recent years, with an emphasis on non-pharmaceutical alternatives to long-term opioid use [14]. Acupuncture [15], hypnosis [16], transcutaneous electrical nerve stimulation [17], and auriculotherapy [18] have each shown promising results as non-pharmaceutical alternatives to long-term opioid use. Additionally, the lack of side effects associated with these complementary approaches makes them increasingly tempting options [19]. However, a major drawback of these complementary medicine approaches is the training required for practitioners to safely and effectively provide these services. For example, eleven states and the District of Columbia require 200–300 h of training for physicians to become acupuncturists, and three states require physicians to obtain a separate acupuncture license [20]. The use of nanoparticles as adjuvants to preexisting analgesics that clinicians are already qualified to prescribe would allow clinicians to avoid the hours of training required to provide complementary medicine alternatives [21]. Nanotechnology is therefore uniquely suited to address not only the concerns associated with long-term opioid use, but also the relative impracticality of implementing complementary medicine practices that require extensive practitioner training.

## 3. Physiology of Pain

**Figure 1:**
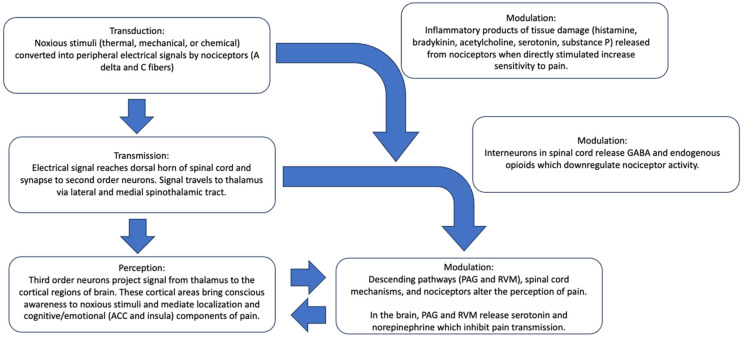
Pain physiology and the four phases of nociception.

Pain is a fundamental yet complex aspect of human physiology, deeply rooted in our evolutionary timeline as an essential component for survival and adaptation to our surroundings [22]. Figure 1 depicts how pain physiology reveals an intricate network of biological processes which involve many different systems within the body. In medicine, pain is one of the most common complaints, and its character of presentation helps give clues to the underlying disease process causing pain [22]. Understanding the pathophysiology of pain is critical to determining its significance in a patient’s health as well as determining future novel treatment options for alleviating pain.

When pain becomes part of the conscious experience, it is referred to as nociception [23]. Just as there are many nerve endings through our skin, viscera, muscles, and organs to detect changes in homeostasis, pain is detected throughout the body by highly specialized sensory neurons developed to be sensitive to noxious stimuli. Nociceptors are the free nerve endings which receive and convert noxious stimuli to nerve impulses to the brain through the spinal cord [23].

The four phases of nociception are transduction, transmission, perception, and modulation. Noxious stimuli in the forms of thermal, mechanical, or chemical stimuli are transduced by nociceptors, or converted into peripheral electrical activity that is transmitted centrally to the spinal cord, brainstem, thalamus, and cortex [24]. Nociceptors come in two primary forms, A delta and C fibers, both with different characteristics that help differentiate the variety of pain impulses brought to conscious awareness. The A delta type represents myelinated and large, fast-conducting fibers which respond to heat and mechanical disruption and are responsible for sharp and immediate pain. These fibers are associated with spinal reflex withdrawal before a pain sensation can be perceived. C fibers, smaller and unmyelinated, are situated in the skin, organs, and tendons, and are the most abundant peripheral nociceptors. They are responsible for the transmission of dull and aching pain, often poorly localizable and constant, and experienced after initial injury [24]. Fast sodium channels, or voltage-gated sodium channels in nociceptors are the main mechanism of transduction in these fibers [25]. They allow the rapid influx of sodium ions which lead to depolarization and the generation of action potentials that transmit pain signals from the periphery to the central nervous system. Local anesthetics, such as lidocaine and bupivacaine, target these channels, blocking the influx of sodium into the neuron. This inhibits depolarization and the propagation of actions potentials, which leads to the loss of sensation in the targeted area and provides the highly effective analgesia for which local anesthetics are known [25]. The transmission of both fibers ends in the dorsal horn of the spinal cord and continues via second-order neurons via the lateral and medial spinothalamic tract to different areas of the thalamus. From here, third order neurons project to different cortical areas of the brain that mediate localization, perception, and cognitive/emotional components of pain [26].

Perception, the third process of nociception, is the cortical interpretation of these afferent stimuli, bringing conscious awareness to pain. Interpretation of pain is a highly individual and variable process, influenced by factors such as genetics, physical and mental health, past pain experiences, age, culture, and even gender roles [27]. The emotional and cognitive aspects of pain are processed in areas such as the anterior cingulate cortex (ACC) and the insula. These regions contribute to the affective and evaluative aspects of pain, influencing how it is experienced [28]. For instance, the ACC is involved in the emotional response to pain, such as distress or anxiety, while the insula is crucial for integrating pain with emotional and cognitive processes [29].

Pain modulation refers to the body’s ability to alter the perception of pain. This process involves both descending pathways from the brain to the spinal cord and local spinal cord mechanisms. The descending pathways originate in areas such as the periaqueductal gray matter (PAG) and the rostroventral medulla (RVM). These areas release neurotransmitters such as serotonin and norepinephrine, which inhibit pain transmission at the spinal cord level [30]. The spinal cord features a host of inhibitory interneurons that can downregulate pain signals. These interneurons release neurotransmitters such as gamma-aminobutyric acid (GABA) and endogenous opioids, which decrease the activity of pain-transmitting neurons [31]. The balance between excitatory and inhibitory signals determines the final perception of pain. Peripherally, products of tissue damage and inflammation, collectively known as “inflammatory soup”, play an important role in the modulation of pain. When nociceptors are stimulated directly, they release chemicals that can activate more nociceptors, increasing sensitivity to pain. These chemicals include histamine, bradykinin, acetylcholine, serotonin, and substance P [24,32]. Central sensitization can also have a modulatory role in persistent, pathological pain conditions that take place following acute injury. In central sensitization, there can be an increased responsiveness in spinal cord transmission neurons, which can often be reproduced by the stimulation of non-nociceptive primary sensory fibers [32].

These modulatory mechanisms and the intercommunication between the central and peripheral nervous systems play a crucial role in our perception of pain and how it is treated in a medical setting. Advances in pain research continue to enhance our understanding of pain physiology and improve pain management strategies. Research into genetic, molecular, and neurobiological mechanisms holds promise for developing targeted therapies for both acute and chronic pain conditions.

## 4. Review of Common Analgesics

### 4.1. NSAIDS

Table 1 reveiews commonly used analgesics, their rout of administration and any associated side effects. Nonsteroidal anti-inflammatory drugs (NSAIDs) are widely used for their antipyretic, analgesic, and anti-inflammatory effects. They are effective for various acute and chronic pain conditions including muscle pain, mild trauma, headaches, and inflammatory conditions such as arthritis [33]. Their primary mechanism of action is via the inhibition of the enzyme cyclooxygenase (COX). COX is thought to be involved in prostaglandin production, which mediates vasodilation. COX-1 is constitutively expressed in the body, and is involved in platelet aggregation, renal function, and the maintenance of the protective gastric mucosa, while COX-2, its isozyme, is expressed only as part of the inflammatory response [34]. Certain NSAIDs (ibuprofen and naproxen) are non-selective COX inhibitors, targeting both COX-1 and COX-2, while others (celecoxib) selectively inhibit COX-2 [8]. NSAIDs have known side effects on the gastrointestinal, cardiovascular, and renal systems. Non-selective COX inhibitors are thought to have a higher risk of GI side effects, including GI bleeds, obstruction ulcers and perforations [33,34]. Cardiovascular side effects include an increased risk for cardiovascular adverse events, such as myocardial infarction or stroke, and are more associated with selective COX-2 inhibitors [33]. Renal side effects are more likely to occur in individuals with underlying renal dysfunction and include electrolyte imbalances and nephrotic syndrome [34].

### 4.2. Acetaminophen

Acetaminophen is another non-opioid analgesic used for its analgesic and antipyretic effects. Alongside NSAIDs, it is recommended as the first step in the WHO analgesic ladder [42]. Acetaminophen is thought to exert its action by being metabolized into p-aminophenol, which can cross the blood–brain barrier and mediate the activity of receptors involved in nociception. Hence, the pain modulation mechanism has effects directly on the brain [35]. When used short-term and within recommended dosages, acetaminophen is generally well-tolerated; however, liver toxicity may occur with high dosages [8,35]. Other side-effects may include rash, nephrotoxicity, and electrolyte abnormalities. Skin reactions may include Stevens-Johnson syndrome, a potentially fatal condition [43].

### 4.3. Gabapentinoids

Gabapentin is an anticonvulsant that is also approved for the treatment of postherpetic neuralgia. It is additionally used off-label for postoperative analgesia and diabetic neuropathy. Gabapentin is an analog of the inhibitory neurotransmitter, gamma-amino-butyric acid (GABA), though it does not act directly on GABA receptors in the CNS. The mechanism of action is thought to be related to its binding of presynaptic voltage-gated calcium channels (specifically the α2δ subunit), inhibiting excitatory neurotransmitter release [36]. Pregabalin, which is similarly an anticonvulsant used for neuropathic pain, works via the same mechanism as gabapentin but has greater potency for the α2δ subunit [8]. Patients with postherpetic neuralgia and diabetic neuropathy taking gabapentin reported substantial benefit and pain relief [44]. Pregabalin is also similarly effective at reducing pain [45]. Both anticonvulsants are associated with drowsiness and somnolence, and withdrawal symptoms may occur after the abrupt discontinuation of the medication [36,44,45].

### 4.4. Antidepressants

Tricyclic antidepressants (TCAs) are a class of medications used for the treatment of major depressive disorders. TCAs work by inhibiting the reuptake of various neurotransmitters, including serotonin and norepinephrine. TCAs are also believed to act on cholinergic, muscarinic, and histamine receptors. These mechanisms are thought to be responsible for their use as an antidepressant and their off-label use in the management of chronic pain [9]. Commonly used TCAs include amitriptyline, doxepin, and imipramine. Although effective for various types of pain, their predominant use is for neuropathic pain [37,40]. Side effects include constipation, weight gain, dizziness, dry mouth, orthostatic hypotension, and urinary retention. TCAs are also associated with cardiovascular side effects including arrhythmias [9,37,40]. Orthostatic hypotension is a significant side effect to consider, as it can lead to falls and fractures, especially in the elderly. TCAs have been associated with an increased risk of hip fractures [37].

Serotonin and norepinephrine reuptake inhibitors (SNRIs) and selective serotonin reuptake inhibitors (SSRIs) also work by inhibiting the reuptake of various neurotransmitters. SNRIs work predominantly by the reuptake inhibition of serotonin and norepinephrine, while SSRIs exert their effects via the inhibition of serotonin reuptake [40]. SNRIs, including duloxetine and venlafaxine, are effective at reducing neuropathic pain, specifically diabetic neuropathy [8,40]. The evidence for SSRIs in the use of pain is less clear, and data regarding their efficacy is not clear, suggesting a modest improvement at best [37]. The side effect profile of SNRIs and SSRIs is better compared to TCAs, however insomnia, anxiety, and GI disturbances are reported among patients on SSRIs [46]. Among the SNRIs, duloxetine is associated with nausea, fatigue, and vomiting, while venlafaxine is associated with a dose-dependent increase in blood pressure [37,40].

### 4.5. Anticonvulsants

Anticonvulsants like carbamazepine and phenytoin have been used to treat trigeminal neuralgia and other neuropathic pain disorders [47]. However, sleepiness, vertigo, nausea, double vision, and reduced neuromuscular control, as well as cognition issues, are the most frequent patient-reported side effects of these drugs, particularly in females [38]. Approved for the treatment of seizures, lamotrigine is also used off-label for the management of fibromyalgia, neuropathic pain, and trigeminal neuralgia [48]. Lamotrigine acts on voltage-gated sodium channels, inhibiting them and preventing the release of presynaptic glutamate. Nausea, vomiting, headache, constipation, and blood dyscrasias are among the side effects of lamotrigine. Skin rashes are also common among patients taking lamotrigine, with Stevens-Johnson syndrome being the most feared and severe presentation [48]. In a review looking at lamotrigine as a treatment for neuropathic pain, nearly 10% of participants reported a skin rash, while the treatment effect was minimal [49].

### 4.6. Local Anesthetics

Local anesthetics are commonly used to help reduce pain associated with medical procedures but can also be used as analgesics for patients with acute and chronic pain. Local anesthetics include esters (procaine, chloroprocaine, tetracaine) and amides (lidocaine, bupivacaine, prilocaine), with the two classes differing in the mechanism of their metabolism [50]. The mechanism of action includes the inhibition of voltage-gated sodium channels, preventing the propagation of nerve signals locally [8]. Local anesthetics can be used topically (as ointments or patches), or injected as a nerve block to help reduce muscle, joint, and nerve pain. Injections may offer patients longer symptomatic relief and more targeted therapy. One concern for the use of local anesthetics in pain management is their relatively narrow therapeutic window [39]. Local anesthetic toxicity can result in arrhythmias, hypertension, agitation, seizures, and respiratory arrest [51]. Considering cyclobenzaprine is an example of a tricyclic antidepressant-like medication, its side effects are similar to the side effects of other TCAs and include dizziness, confusion, dry mucus membrane, and urinary retention [45,48].

### 4.7. Muscle Relaxants

Muscle relaxants describe a broad class of pharmacologic agents used to induce the relaxation of skeletal muscles. They include drugs with various mechanisms of action and therefore a wide range of side effects [52]. Baclofen is an example of an antispastic medication used for spasticity, seen in patients with spinal cord lesions, as well as off-label for trigeminal neuralgia [53]. Baclofen has also been shown to be effective in reducing pain symptoms in patients with low-back pain [54]. Baclofen acts as an agonist of the GABA-B receptor located in the brain and spinal cord, hence stimulating inhibitory neuronal transmission, promoting the relaxation of spasticity [53]. Cyclobenzaprine is also used for muscle spasticity and works similarly to TCAs. In addition to its use as a muscle relaxant it can work to reduce pain in patients with fibromyalgia and neuropathic pain [55]. Baclofen’s most significant side effects occur when the medication is abruptly stopped. Without gradual tapering of the medication, patients may experience withdrawal symptoms such as hallucinations, muscle rigidity, seizures, and organ failure [52,53]. Considering cyclobenzaprine is an example of a tricyclic antidepressant-like medication, side effects are similar to the side effects of other TCAs and include dizziness, confusion, dry mucus membranes, and urinary retention [52,55].

## 5. Why Nanotechnology

In addition to the previously discussed adverse side effects associated with conventional analgesic methods, the inability for target therapy also represents a significant challenge in achieving long-term pain relief [56]. Nanoparticles are uniquely positioned to address this problem because they have design parameters that traditional drugs do not; for example, size, shape, and surface charge can all be optimized to create nanomaterials that can target specific tissues or subcellular organelles [57]. Alterations in the activity of the NMDA receptor are critical in the pathogenesis of chronic pain. The activation of the NR2B subunit of the NMDA receptor alters ion permeability, inducing further NMDA receptor activation through chronic glutamate release and the subsequent excitatory postsynaptic potentials (EPSPs) [58]. EPSPs induce further separation of Mg2+ from the NR2B subunit of NMDA receptors, further inducing EPSPs and exacerbating chronic pain [58]. Using hydroxyapatite nanomaterials, which are biocompatible and have been approved for use in bone-related diseases, Gu and colleagues encapsulated and efficiently delivered NR2B-siRNA into mice via intrathecal injection [59]. Immunohistochemical staining of spinal cord segments showed a decrease in the number of cells expressing the NR2B protein, thus demonstrating the ability of nanoparticles to facilitate the inhibition of NR2B expression [59]. Nanoparticles therefore represent an opportunity to directly target the mechanisms of chemical plasticity that contribute to chronic pain.

Another challenge associated with long-term opioid use is that conventional administration leads to the uncontrolled release of the drug [60]. This leads to a significant fluctuation in drug plasma concentration, with the amount of drug oscillating between sub-therapeutic concentrations and potentially lethal doses [60]. Nanoparticulate systems have been developed to address this problem. DepoFoam^TM^ is a nanotechnology that facilitates a reduction in dosage frequency by providing a consistent concentration of analgesic throughout the entire treatment period [61]. It does so via the encapsulation of drugs into multivesicular liposomes, which can facilitate targeted delivery, thereby improving the bioavailability and stability of drug molecules in the body [61]. Maintaining consistent concentrations of the drug in the body is critical not only for reducing overdose risk, but also for facilitating the more accurate dosing for patients with chronic kidney disease (CKD). Opioids are primarily cleared in the urine, but the effect of renal failure and age-related reductions in GFR on safe and effective opioid dosing is not well understood [62]. Because nanoparticles can facilitate the more consistent and predictable release of analgesic medications, they have tremendous potential in the creation of accurate dosage adjustments for patients with renal impairment.

## 6. Current Nanoparticulate Drug Delivery Systems

Using nanotechnology, there has been extensive development in the field of drug delivery, yielding several nanomaterials approved for clinical use [63]. Nanomaterials can be efficiently loaded with medications [64], which helps protect the stability of protein-based drugs [65] and sustain their controlled release by prolonging circulation time. Nanomaterials have been used to encapsulate free molecules and protein-based drugs, increasing blood circulation time and resulting in longer-lasting pain relief while minimizing side-effects [57]. Nanomaterials already approved by the FDA include liposomes [66], Poly Lactic-co-Glycolic Acid (PLGA) [67], and other carbon-based polymer nanomaterials.

### 6.1. Nanotechnology-Based Transporters (Liposomes)

Liposomes are derived from cellular-like lipids, making them biocomparable. PEGylated liposomes have successfully encapsulated and enhanced the accumulation of zoledronic acid (ZOL), an inhibitor of the ras-dependent Erk-mediated pathway. This liposomal-based drug delivery system has passed across the blood–brain barrier, promoting the release of ZOL for the treatment of neuropathic pain [68]. Liposomes have also been used to encapsulated hydromorphone, whose delivery prolonged pain relief in rats with a single injection [69].

In a clinical trial, morphine, or DepoDur^TM^, was combined with a liposome-based formulation, DepoFoam^TM^. Patients were randomized to receive a single dose of DepoDur^TM^ or placebo. All of the DepoDur^TM^ doses reduced patient-controlled fentanyl use and significantly improved pain control at rest through 48 h, both at rest and during activity, compared to the placebo and conventional epidural morphine [70].

A recent liposomal preparation, Zynrelef^TM^, combining bupivacaine and meloxicam has been used to treat acute pain [63]. The meloxicam in this formulation has been found to increase the effectiveness of bupivacaine by maintaining the pH of the preparation which is applied during wound closure [63]. The viscous solution Zynrelef^TM^ can be applied at the level of the subcutaneous tissue and/or at the soft tissue incision level and has been approved by the FDA for abdominal surgery, open hemorrhoidectomy, total knee arthroplasty, and bunionectomy at the time of closure. In two phase III clinical studies, Zynrelef^TM^ was compared to bupivacaine HCl and placebo for both patients receiving bunionectomy with osteotomy and internal fixation under regional anesthesia [71], as well as unilateral open inguinal herniorrhaphy with mesh placement under general anesthesia [72]. In both trials, Zynrelef^TM^ was associated with significantly reduced postoperative pain and opioid consumption up to 72 h postoperatively compared to plain bupivacaine. This formulation has since been extended to include open shoulder and spine surgery in 2024.

### 6.2. Polymeric Nanoparticles

Polymeric nanoparticles’ main characteristics are biocompatibility, biodegradability, and non-toxicity [73]. Poly lactic-co-glycolic acid (PLGA) is one of the most successful biodegradable polymers and has been commercialized for a variety of drug delivery systems [74]. Its hydrolysis leads to metabolite monomers, lactic acid, and glycolic acid, which can be easily metabolized by the Krebs cycle and are thus associated with minimal systemic toxicity [75]. Bupivacaine has been nanoencapsulated by PLGA to prolong its release and improve pain relief [76]. PLGA nanoparticles have been used to encapsulate synthetic cannabinoid CB13 in a murine neuropathic pain model, which helped achieve an analgesic effect for up to 11 days [77]. Likewise, PLGA nanomaterials have been used to encapsulate p38 siRNA to enhance its stability and slow its release [78]. In addition, p38 siRNA nanoparticles reduced mechanical allodynia and microglial activation in rats, downregulating the expression of p38-related inflammatory genes, such as TNF-α [78].

Apart from prolonging pain relief, PLGAs have also been used to attenuate side effects. The sodium and calcium channel blocker lamotrigine has been demonstrated to be an effective treatment for neuropathic pain in multiple randomized controlled trials [79]. However, its clinical applications have been limited due to its risk of dose-dependent rashes, secondary to its non-selective distribution to organs other than the brain [80]. Lamotrigine-carrying PLGA nanoparticles were shown to preferentially distribute these nanoparticles to the brain and reduce accumulation in non-target organs in a mouse model for partial sciatic nerve injury [79].

PLGA nanoparticles have been used to treat bone pain. Between 70 and 90% of patients with bone metastases experience chronic pain [81]. Often untreatable and associated with intractable pain, Alendronate-conjugated PLGA-Cabazitaxel nanoparticles have been developed [82]. Mice bone tumor models showed lower pain and reduced tumor burden, and an improved maintenance of bone structure compared to the free-drug treated control group [82].

Nanoparticle drug delivery provides many advantages because of their highly stable nature and ability to encapsulate different active ingredients [73]. Biodegradable synthetic polymers, such as poly-lactic acid (PLA), poly-glycolic acid (PGA), poly-hydroxybutyrate (PHB) and the aforementioned PLGA, have been approved by the US Food and Drug Administration and the European Medicine Agency [83]. These polymers have confirmed biocompatibility, and low levels of toxicity and biodegradation in in vivo studies [84].

### 6.3. Nanotechology-Based Devices and Patches

Nanocapacitor patches have been used to relieve acute and chronic pain in specific body locations, typically related to arthritis, neuropathy, radiculopathy, myofascial, or musculoskeletal pain [10]. NeuroCuple^TM^ is a nanocapacitor device/patch which uses capacitive coupling to allow the transfer of electrical energy without a direct electrical connection. When used in patients receiving a unilateral total knee or total hip arthroplasty, NeuroCuple^TM^ was associated with a significant 34% reduction in pain at rest during postoperative days 1–3, as well as reducing the number of patients requesting an opioid prescription following hospital discharge by 52% [12]. It is suggested that after local trauma, the cellular membranes which ordinarily play a role in the electrical equilibrium are destroyed, resulting in an accumulation of electrons. This causes a decrease in pH and the accumulation of fluid which causes local inflammation and pain [85]. The local application of NeuroCuple^TM^ allows the redistribution of the local excess of electrons, normalizing the local pH and reducing inflammation/pain [12].

### 6.4. Enhanced Drug Targeting

Most nanoparticle drug delivery systems (NDDSs) attempt to prolong a medication’s duration of action and therapeutic effect. However, many NDDSs use external stimuli to allow drug release on demand. Possible stimuli include light, heat, ultrasound, the magnetic field, and the electric field to reduce opioid use and minimize side effects [86].

Light-activated NDDSs use varying spectrums of light to enable drug administration [87]. Near-infrared (NIR) light-triggered liposomes have been loaded with Tetrodotoxin and photosensitizer to deliver adjustable local anesthesia to a rat sciatic nerve after irradiation at 730 nm [88]. NDDSs have also been combined with the photothermal effect of Copper Sulfide to achieve the repeated on-demand release of Bupivacaine after NIR excitation [89]. NIR-triggered NDDSs have similarly been used in patient-controlled transdermal microneedles. During irradiation, lidocaine can be delivered through an implanted microneedle which enters the bloodstream within 10 min [90]. A current limitation of light-activated NDDSs includes the varying depths of light penetration from patient to patient, likely secondary to hair, tissue thickness, and tissue type [91].

Ultrasound, which has deeper tissue penetration than NIR, has been used as a trigger for on-demand local anesthesia [91]. In one study, ultrasound insonation was used to encapsulate a sonosensitizer protoporphyrin IX, producing reactive oxygen species which react with the liposomal membrane and leading to Tetrodotoxin (TTX) release the production of a nerve block which lasted over 8 h in rats. Subsequent insonation reproduced nerve blocks twice more at 0.7 and 0.2 h [91]. Ultrasound has also been used with poly vanillyl alcohol-co-oxalate nanoparticles to scavenge H_2_O_2_ and exert anti-inflammatory and antioxidant effects in musculoskeletal injuries in mice [92]. The nanoparticles suppressed proinflammatory cytokines TNFα and IL-1β, and increased VEGF and PECAM-1 levels, improving blood perfusion to the damaged tissues [93].

Magnetic nanoparticles (MNPs) have been used in both animals and humans for the targeted delivery of chemotherapeutics [94]. IV injections of MNP complexes with ropivacaine in rats and subsequent magnet applications at the ankle have been used to improve anesthesia to mirror ankle blocks in humans [95]. The plasma concentration of the complexed ropivacaine was greater when compared to direct drug injection [95].

## 7. Patents Update

Of the aforementioned nanoparticulate drug delivery systems, several have been patented for clinical use. Pacira Pharmaceuticals filed a patent for DepoDur^TM^ in 1997, the liposomal encapsulation which facilitates the slow release of morphine via the manipulation of the neutral lipid molar ratios; the patent was granted in 1999 and ultimately expired in 2017 [96]. Pacira Pharmaceuticals also filed a patent for DepoFoam^TM^, another liposomal encapsulation with an adjustable size designed to facilitate controlled release, in 1998; the patent was granted in 2000 and expired in 2013 [97]. Heron Therapeutics filed a patent for Zynrelef^TM^, the liposomal formulation of bupivacaine and meloxicam designed to promote pH stability, in 2017; the patent was granted in 2019 and remains active [98]. A patent was filed by nCAP Licensing for NeuroCuple^TM^, the nanocapacitor-based pain relief patch, in 2020, and it was ultimately granted in 2022 [99]. PLGA drug delivery systems have not yet been patented for analgesic purposes, but the University of North Texas Health Sciences Center was granted a patent in 2015 for targeted cancer chemotherapeutics utilizing the technology [100]. Table 2 summarizes the nanoparticulate systems currently patented for use in pain management.

## 8. Clinical Trials Update

Several clinical trials have demonstrated the ability of nanoparticulate systems to serve as drug delivery systems in the ongoing efforts to provide effective postoperative analgesia while avoiding the adverse effects associated with opioid use. DepoFoam^TM^ bupivacaine is a novel liposomal formulation of bupivacaine designed to provide prolonged postsurgical analgesia [61]. One clinical trial randomized 138 patients undergoing total knee arthroplasty to receive either DepoFoam^TM^ bupivacaine or bupivacaine HCl; results showed significantly lower pain intensity scores on postoperative days 1–5 and significantly higher rates of care provider satisfaction with analgesia for the DepoFoam^TM^ group [101]. Another clinical trial enrolling 140 patients undergoing total knee arthroplasty demonstrated superior safety metrics for patients receiving liposomal bupivacaine compared to bupivacaine HCl; in addition to significantly lower visual analog pain intensity scores, patients in the liposomal bupivacaine group had a significantly lower mean opioid consumption 48 h after surgery (18.7 mg vs. 84.9 mg, *p* = 0.0048), significantly longer times to the first opioid rescue, and a significantly higher percentage of opioid-free patients [102]. Another clinical trial randomized 41 patients to receive either liposomal bupivacaine or bupivacaine HCl after distal radius fracture repair; patients in the liposomal bupivacaine group reported significantly lower pain levels on the day of surgery and consumed significantly lower numbers of opioid pills and oral morphine equivalents [103].

Liposomal bupivacaine also showed superior analgesic efficacy in patients undergoing laparoscopic or robotic-assisted hysterectomy. A clinical trial randomized 56 patients to receive either liposomal bupivacaine or bupivacaine HCl and showed that patients in the liposomal bupivacaine group had significantly lower average postoperative pain scores as well as decreased worst pain scores on postoperative days 2 and 3 compared to patients who received bupivacaine HCl [104]. Liposomal bupivacaine also showed several benefits in patients undergoing implant-based breast reconstruction. A clinical trial enrolling 24 patients demonstrated significantly lower opioid and benzodiazepine consumption, shorter hospital stays, and lower average healthcare costs ($10,828 vs. $18,632, *p* = 0.039) for patients receiving liposomal bupivacaine compared to 0.25% bupivacaine with epinephrine [105]. Liposomal bupivacaine’s demonstrated ability to provide superior analgesia as well as reduce opioid consumption and healthcare costs, makes it a promising candidate for postoperative analgesia in the continuing efforts to avoid the adverse effects associated with opioid use.

In addition to the promising results of clinical trials evaluating the efficacy of liposomal bupivacaine, the NeuroCuple^TM^ patch has demonstrated promising results in a randomized controlled open-label study, with a significant reduction in pain on postoperative days 1–3 and significantly lower rates of patients requesting opioid prescriptions upon discharge for patients receiving analgesia with NeuroCuple^TM^ compared to the standard of care [12]. While these findings are encouraging, NeuroCuple^TM^ represents an avenue for further investigation via additional placebo-controlled studies evaluating its efficacy. PLGA-based drug delivery systems, with a demonstrated potential to serve as encapsulations that facilitate the extended release of drugs, such as naproxen and celecoxib, also require further investigation via clinical trials, as current research is still in the preclinical stage [106,107]. Table 3 summarizes clinical trials evaluating the efficacy of nanoparticulate systems in postoperative analgesia.

## 9. Future Ongoing Work

### 9.1. Detection of Pain Biomarkers

There has been ongoing work investigating the feasibility of nanoparticles to localize neural pathology lesions which may cause neuropathic pain. Diabetes mellitus commonly causes neuropathy and is a common form of chronic pain, though questionnaires and diagnostic tests are often unable to localize the source of a patient’s pain [8]. One rat study showed that MMPs are upregulated after nerve injury and may show elevated levels for up to 20 days as a part of the neuroinflammatory process. Magnetic IONPs were used to target MMP-12 in spinal nerve ligations. MRI scans were subsequently taken, which showed a significant uptake at the lesions [108]. Other proteins such as aquaporin-4, interleukin-1, and periaxin may also be considered for locating lesions [8].

### 9.2. Vectors for Delivery of Gene Therapy

#### 9.2.1. Viral Vectors

**Figure 2:**
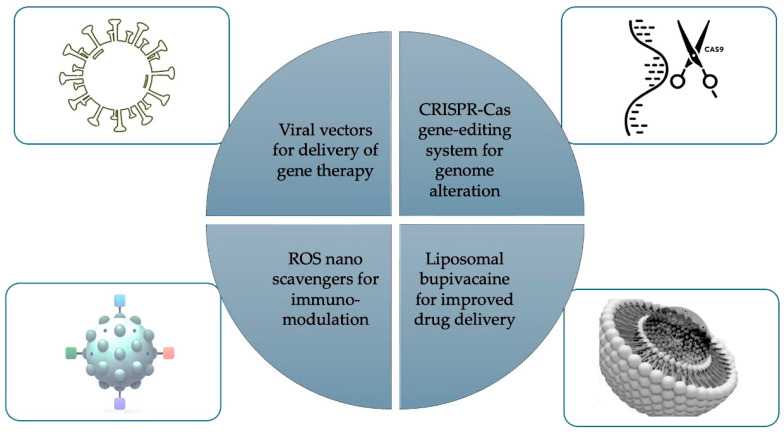
Innovative nanotechnologies.

Figure 2 shows some innovative nanotechnolgies being studied, including viral and non-viral vectors, CRISPR, Liposomal bupivacaine and ROS nanoscavengers. Gene therapy may allow the specific targeting of a disease or gene and localized delivery to a pain source. Chronic pain treatment could include reducing nociception by inducing the overexpression of analgesic genes and anti-inflammatory cytokines [109]. On the contrary, pain producing genes could be inhibited. Safe viral vectors, such as for the transfer of therapeutic gene materials have been researched to target non-dividing cells, such as neurons [110]. Viral vector options currently include herpes simplex virus type 1 (HSV-1), Adeno-associated viruses (AAVs), Adenoviruses (AVs), and Lentiviruses (LVs) [109]. HSV-1 may be an ideal carrier for pain treatment given its high packaging capacity and innate neurotropism, which allows its use in dermal application or subdermal injection. AAVs have been used to deliver genes in mice via intrathecal injections, which then target and trigger neuronal cells to secrete opioid-like proteins in low and sustained amounts [109].

HSV vectors expressing ENK and PENK have demonstrated anti-nociceptive, anti-neuropathic, and anti-inflammatory effects in in vivo models and may improve the ability to release endogenous opioids [111]. HSV vectors expressing the glycine receptor (GlyR) have been shown to function as an endogenous opioid [112]. In another study, HSV vectors were used to express IL-10 in a Type I diabetes rat model by reducing the expression of Toll-like receptor 4 (TLR4), which reduced macrophage activation and inhibited painful neuropathy [113].

#### 9.2.2. Non-Viral Vectors

Many non-viral vectors have also been used to treat pain. Generally, non-viral vectors are less immunogenic, safer, and more stable than viral vectors [114]. Some non-viral vectors are cationic lipids and polymers, plasmids, naked DNA, and lipid-polymers. IL-2 expressing plasmids have been administered via a spinal catheter in rat models to demonstrate a dose-dependent pain reduction [115]. In another study, naked IL-10-encoded plasmids with D-mannose administered via a single intrathecal injection in rats showed stable long-term neuropathic pain relief [114]. Another example of non-viral gene transfer was used to help prevent cisplatin-induced neuropathy. Cisplatin has been shown to cause a dose-dependent neuropathy [116]. Neurotropin-3 (NT-3)-encoded plasmids were injected intramuscularly and were followed by electroporation with four square-wave pulses in a cisplatin-treated mouse model. Sensory distal latencies were lower in the treated rats, indicating that neuropathic pain was reduced [116].

### 9.3. CRISPR-Cas

CRISPR-Cas is a gene-editing system which allows particular locations in the genome to be added, deleted, or altered. CRISPR may be used to modulate gene expression and reduce proinflammatory signals. Using lentiviral vectors, researchers were able to inhibit NF-κB activation and reduce inflammation in vitro [117].

In one study, CRISPR-Cas9 was investigated as a possible treatment tool for osteoarthritis [118]. AAVs expressing CRISPR-Cas9 were used to target NGF, IL-1β, and MMP13, which are commonly upregulated in osteoarthritis. Shutting off NGF, IL-1β, and MMP13 both reduced pain and inhibited disease progression in a surgical mouse model [118].

Nanoparticles, rather than viral vectors, have been researched as a CRISPR-Cas delivery system [119]. CRISPR-Gold is a specialized nanoparticle carrier which has been used to target neurons and muscle cells. Advances in CRISPR may eventually develop a platform to monitor a patient’s pain and inflammation and allow gene expression modulation to reduce them to health levels [119].

### 9.4. Scavengers

When in acute pain, unwanted immune activation and central sensitization can both worsen pain and cause chronic pain [120]. Scavengers are immunomodulatory nanomaterials specifically designed to proactively remove those overproduced immune agents and thus reduce pain and inflammation [121]. Two notable scavengers are nucleic acid-binding scavengers (NABS) and reactive oxygen species (ROS) scavengers.

Danger and pathogen-associated molecular patterns (DAMPs and PAMPs) stimulate toll-like receptors (TLRs) to activate an innate immune response [120]. In chronic disease, however, DAMPs and PAMPs can be overstimulated in healthy cells. NABS are charged polymers and nanoparticles are used to reduce TLR overstimulation, reducing pain and inflammation [122]. Rather than treating symptoms of pain, scavengers eliminate the cause of the pain by preventing TLR overexpression.

ROS are byproducts of oxidative phosphorylation and aid in cell signaling and pathogen defense [121]. Excess ROS may be present in inflammatory states, creating a state of high reactivity and a reduced pain threshold. ROS scavengers naturally remove ROS that haven’t been metabolized. Scavenging excess ROS may increase the threshold for pain and reverse central sensitization. Current ROS scavengers are nonspecific and can be cytotoxic [123]. Nanoparticles have been used to scavenge ROS. However, nanoscavengers have low motility and cannot reach some intracellular locations. Clinical research is being conducted to improve ROS scavengers’ efficiency and biocompatibility. One study is using hemin to improve the motility of mesoporous silica nanoparticles (MSNs) by altering the thickness of the nanoparticle shell [123]. Another study added ultrasmall ceria nanocrystals to MSN to create a nanocomposite which may better localize ROS scavenging and improve wound repair [124]. Table 4 highlights selected nanoformulations which have been explored in animal models.

## 10. Conclusions

Nanotherapeutics have just begun to be explored in the field of pain medicine. Chronic pain is associated with many diseases, many that are quite difficult to treat, and costs the U.S. healthcare system substantially. Many of the current therapeutics provide inadequate relief and are associated with various side effects. Nanotechnology offers an innovative way to address the pain that millions are suffering from. Nanomaterials can be designed to distinguish locations of pain, target specific tissues, modulate key pain pathways, and maintain consistent drug concentrations, addressing some of the limitations of conventional analgesics. Liposomal preparations for already existing treatments for pain have both improved and extended their duration of action. PLGA nanomaterials have been researched for their ability to extend the duration of medication effectiveness while minimizing side effects. Nanocapacitor patches have been used to treat both acute and chronic pain and offer a non-invasive approach to pain management. Gene therapy, using both viral and non-viral vectors, has been investigated as a method to deliver more effective and longer-lasting pain relief. Additionally, CRISPR may be able to modulate gene expression to reduce a patient’s pain and inflammation, offering more individualized analgesic management strategies compared to current pain treatment modalities. Lastly, scavengers may be able to reduce pain and pain sensitization via molecules like free nucleic acids and ROS. Despite these great advances in this field, there is still much research that needs to be carried out in order to use nanotechnology in clinical practice. As the field of pain management advances, patients will hopefully gain access to tailored treatments that not only effectively address their pain with fewer side effects but also contribute to reducing dependence on opioids, helping combat the opioid epidemic.

## Figures and Tables

**Table 1:** Common analgesics and associated side effects.

Common Analgesics	Routes of Administration	Side Effects
Opioids	Oral (most common), intramuscular, intravenous, intrathecal, rectal, subcutaneous	Constipation, nausea, drowsiness, fractures, respiratory depression [10]
NSAIDs	Oral (most common), topical, intravenous, rectal	GI bleed, ulcer perforation, myocardial infarction, electrolyte abnormalities, nephrotic syndrome [33]
Acetaminophen	Oral, rectal, intravenous *	Rash (including Stevens-Johnson syndrome), nephrotoxicity, electrolyte abnormalities, liver toxicity [35]
Gabapentinoids	Oral	Drowsiness, somnolence, nausea, dizziness [36]
Antidepressants	Oral (most common), transdermal	Orthostatic hypotension, constipation, weight gain, dizziness, dry mouth, insomnia, anxiety, nausea [37]
Anticonvulsants	Oral (most common), intravenous, intramuscular	Vertigo, nausea, double vision, skin rashes (including Stevens-Johnson syndrome), blood dyscrasias [38]
Local Anesthetics	Topical, intravenous (most common), intrathecal, subcutaneous	Arrhythmias, hypertension, agitation, seizures, respiratory arrest [39]
Muscle Relaxants	Oral (most common), transdermal, intrathecal	Hallucinations, muscle rigidity, seizures, dizziness, confusion, dry mucus membranes [40]

(IV acetaminophen has limited clinical benefits when compared to oral or rectal administration *) [41].

**Table 2:** Nanoparticulate systems patented for pain management.

Nanotechnology	Developer	Description	Clinical Use	Patent Details
DepoDur™	Pacira Pharmaceuticals San Diego, CA, USA	Liposomal encapsulation of morphine for slow release via neutral lipid molar ratio manipulation.	Postoperative pain management	Filed: 1997; Granted: 1999; Expired: 2017 (Willis, 1999) [96]
DepoFoam™	Pacira Pharmaceuticals San Diego, CA, USA	Adjustable size liposomal system for controlled drug release.	Local anesthetics and other drugs	Filed: 1998; Granted: 2000; Expired: 2013 (Sankaram and Kim, 2000) [97]
Zynrelef™	Heron Therapeutics Solana Beach, CA, USA	Liposomal formulation of bupivacaine and meloxicam designed for pH stability and extended analgesia.	Postoperative pain relief	Filed: 2017; Granted: 2019; Active (Ottoboni and Girotti, 2019) [98]
NeuroCuple™	nCAP Licensing Heber City, UT, USA	Nanocapacitor-based pain relief patch that modulates pain signals using electromagnetic fields.	Chronic pain management	Filed: 2020; Granted: 2022 (Spencer and Sutera, 2022) [99]
PLGA-Based Drug Delivery	University of North Texas Fort Worth, TX, USA	Targeted cancer chemotherapeutics using PLGA nanoparticles; potential for future analgesic applications.	Cancer treatment (potential for pain management)	Granted: 2015 (Braden and Vishwanatha, 2015) [100]

**Table 3:** Clinical trials evaluating efficacy of nanotechnology in postoperative analgesia.

Nanotechnology	Study Type	Study Population	Key Findings	References
DepoFoam^TM^ Bupivacaine	Randomized trial in total knee arthroplasty	138 patients	Significantly lower pain intensity scores on postoperative days 1–5; higher care provider satisfaction with analgesia.	Bramlett et al., 2012 [101]
Liposomal Bupivacaine	Randomized trial in total knee arthroplasty	140 patients	Lower visual analog pain intensity scores, reduced opioid consumption (18.7 mg vs. 84.9 mg, *p* = 0.0048), longer time to first opioid rescue.	Mont et al., 2018 [102]
Liposomal Bupivacaine	Randomized trial after distal radius fracture	41 patients	Lower pain levels on surgery day; reduced opioid pill consumption and oral morphine equivalents.	Alter et al., 2017 [103]
Liposomal Bupivacaine	Randomized trial in laparoscopic hysterectomy	56 patients	Lower average and worst pain scores on postoperative days 2 and 3 compared to bupivacaine HCl.	Barron et al., 2017 [104]
Liposomal Bupivacaine	Trial in implant-based breast reconstruction	24 patients	Reduced opioid/benzodiazepine use, shorter hospital stays, lower healthcare costs ($10,828 vs. $18,632, *p* = 0.039).	Motakef et al., 2017 [105]
NeuroCuple™ Patch	Open-label study on postoperative pain	69 patients	Reduced pain on postoperative days 1–3; fewer patients requested opioids upon discharge.	Chelly et al., 2023 [12]

**Table 4:** Nanoformulations explored in animal models.

Nanotechnology	Study Purpose	Key Findings	References
Transfer of ppβEP using self-complementary recombinant adeno-associated virus serotype 8 (sc-rAAV8) vectors	Analgesic gene transfer and expression with IT sc-rAAV8 using an in vivo rat model undergoing L5 spinal nerve ligation	sc-rAAV8 selectively transduced primary sensory neurons in DRG if administered IT, established long term gene expression after single vector administration, leading to significant reversal of allodynia in rats with neuropathic pain by expressing analgesic gene ppβEP.	Storek et al., 2008 [109]
Gene transfer of PVAX1-PENK using plasmid DNA vector	Investigation of anti-nociceptive efficacy of intramuscular and intrathecal delivery of pVAX1-PENK vs. pVAX1 on induced inflammatory pain and SNI neuropathic pain in mice.	Pain thresholds in the pVAX1-PENK-treated mice were significantly higher on day 3, reached a peak on day 7, and lasted until day 28 after gene transfer. pVAX1-treated mice did not significantly improve pain thresholds. Peripheral or spinal delivery of pVAX1-PENK provides a potential therapeutic strategy for inflammatory pain and neuropathic pain.	Hu et al., 2016 [110]
HSV-based vector preproenkephalin transgene product delivery	Assessment of the efficacy of pancreatic surface delivered ENK-encoding HSV-1 on spontaneous behaviors, spinal cord, and pancreatic ENK expression in rats with DBTC-induced pancreatitis.	DBTC/HSV-ENK-treated rats had significantly improved spontaneous exploratory activities, increased met-ENK staining in the pancreas and spinal cord, and normalized c-Fos staining in the dorsal horn. Histopathology of pancreas in DBTC/HSV-ENK-treated rats showed preservation of acinar cells and cytoarchitecture with minimal inflammatory cell infiltrates, compared to controls.	Lu et al., 2007 [111]
HSV-based vector-mediated expression of IL10	Investigation of the continuous delivery of IL10 through HSV vector- mediated transduction in nerve fibers and blockade of nociceptive and stress response in DRG in rats with Type 1 Diabetes.	Reduction in IL1β expression along with inhibition of phosphorylation of p38 MAPK and PKC. Continuous expression of IL10 alters TLR-4 expression in DRG with increased expression of heat shock protein (HSP)-70 in conjunction with the reduction in pain.	Thakur et al., 2016 [113]
Human IL-2 cDNA cloned into pcDNA3 containing a cytomegalovirus promoter	Evaluation of the effect of intrathecal delivery of human IL-2 gene on rat neuropathic pain induced by chronic constriction injury of the sciatic nerve. Paw-withdrawal latency induced by radiant heat used as a measure of pain threshold.	Recombinant human IL-2 had a dose-dependent antinociceptive effect, lasting for 10–25 min. The pcDNA3-IL-2 or pcDNA3-IL-2/lipofectamine complex showed dose-dependent antinociceptive effects, reached a peak on day 2–3 and was maintained for up to 6 days. Liposome-mediated pcDNA3-IL-2 produced a more powerful antinociceptive effect than pcDNA3-IL-2 alone	Yao et al., 2002 [115]
Intrathecal IL-10 gene therapy with co-administration of mannose receptor (MR; CD206) ligand d-mannose (DM)	To evaluate spinal non-viral DM/pDNA-IL-10 co-therapy as a framework for the development of non-viral gene therapeutic approaches for neuropathic pain using wild-type and IL-10 KO mice.	IT application of naked plasmid DNA expressing the IL-10 transgene co-injected with DM (DM/pDNA-IL-10) for the treatment of peripheral neuropathic pain in IL-10 KO mice results in a profound and prolonged bilateral pain suppression.	Vanderwall et al., 2018 [114]
Neurotropin-3 (NT-3)-encoded plasmids	Neurotropin-3 (NT-3)-encoded plasmids injected intramuscularly and followed by electroporation with four square-wave pulses in a cisplatin-treated mouse model in prevention of cisplatin-induced neuropathy.	Sensory distal latencies were lower in the treated rats, indicating neuropathic pain was reduced.	Pradat et al., 2001 [116]
PLGA nanomaterials	PLGA nanomaterials used to encapsulate p38 siRNA to enhance its stability and slow its release in rat model	p38 siRNA nanoparticles reduced mechanical allodynia and microglial activation in rats, downregulating the expression of p38-related inflammatory gene TNF-α.	Shin et al., 2018 [78]
Alendronate-conjugated PLGA-Cabazitaxel nanoparticles	Explore role of PLGA nanoparticles in the treatment of chronic bone pain in the setting of metastases using mice bone tumor models	Mice bone tumor models showed lower pain and reduced tumor burden, and improved maintenance of bone structure as compared to the free-drug treated control group.	Gdowski et al., 2017 [82]

## Data Availability

No new data were created or analyzed in this study. Data sharing is not applicable to this article.
